# RETRACTION: Effect of Open Surgical and Percutaneous Dilatational Tracheostomy on Postoperative Wound Complications in Patients: A Meta‐Analysis

**DOI:** 10.1111/iwj.70344

**Published:** 2025-03-17

## Abstract

**RETRACTION:** MengX. , ShaoY. , and ZhuW. , “Effect of Open Surgical and Percutaneous Dilatational Tracheostomy on Postoperative Wound Complications in Patients: A Meta‐Analysis,” International Wound Journal 21, no. 1 (2024): e14368, 10.1111/iwj.14368.37736875 PMC10788584

The above article, published online on 22 September 2023, in Wiley Online Library (http://onlinelibrary.wiley.com/), has been retracted by agreement between the journal Editor in Chief, Professor Keith Harding; and John Wiley & Sons Ltd. Following an investigation by the publisher, all parties have concluded that this article was accepted solely on the basis of a compromised peer review process. The editors have therefore decided to retract the article. The authors did not respond to our notice regarding the retraction.

**RETRACTION:** X. Meng , Y. Shao , and W. Zhu , “Effect of Open Surgical and Percutaneous Dilatational Tracheostomy on Postoperative Wound Complications in Patients: A Meta‐Analysis,” International Wound Journal 21, no. 1 (2024): e14368, 10.1111/iwj.14368.37736875 PMC10788584

The above article, published online on 22 September 2023, in Wiley Online Library (http://onlinelibrary.wiley.com/), has been retracted by agreement between the journal Editor in Chief, Professor Keith Harding; and John Wiley & Sons Ltd. Following an investigation by the publisher, all parties have concluded that this article was accepted solely on the basis of a compromised peer review process. The editors have therefore decided to retract the article. The authors did not respond to our notice regarding the retraction.

